# A mate to die for? A model of conditional monogyny in cannibalistic spiders

**DOI:** 10.1002/ece3.372

**Published:** 2012-09-13

**Authors:** Lutz Fromhage, Jutta M Schneider

**Affiliations:** 1 Department of Biological and Environmental Sciences, University of JyväskyläP.O. Box 35, 40014, Jyväskylä, Finland; 2 Zoological Institute, University of HamburgMartin-Luther-King-Platz 3, 20146, Hamburg, Germany

**Keywords:** Argiope, dynamic programming, mating strategies, monogamy, monogyny, sexual cannibalism, sexual selection, terminal investment

## Abstract

Monogynous males in various species actively limit themselves to mating with a single female in their lifetime. Whereas previous models have considered monogyny as an obligate mating strategy, here we explore the potential of monogyny to evolve as a context-specific (conditional) behavior. Using a state-dependent dynamic game model based on the biology of the cannibalistic spider *Argiope bruennichi*, we confirm that conditional monogyny can evolve under broad conditions, including an even sex ratio. We predict that males should make a terminal investment when mating with large, virgin females, especially if population density is low and the encounter occurs late in the season. We encourage empirical tests for the existence of conditional monogyny in all species where monogyny occurs in the absence of strict morphological constraints that would make it obligatory.

## Introduction

**Figure 1:**
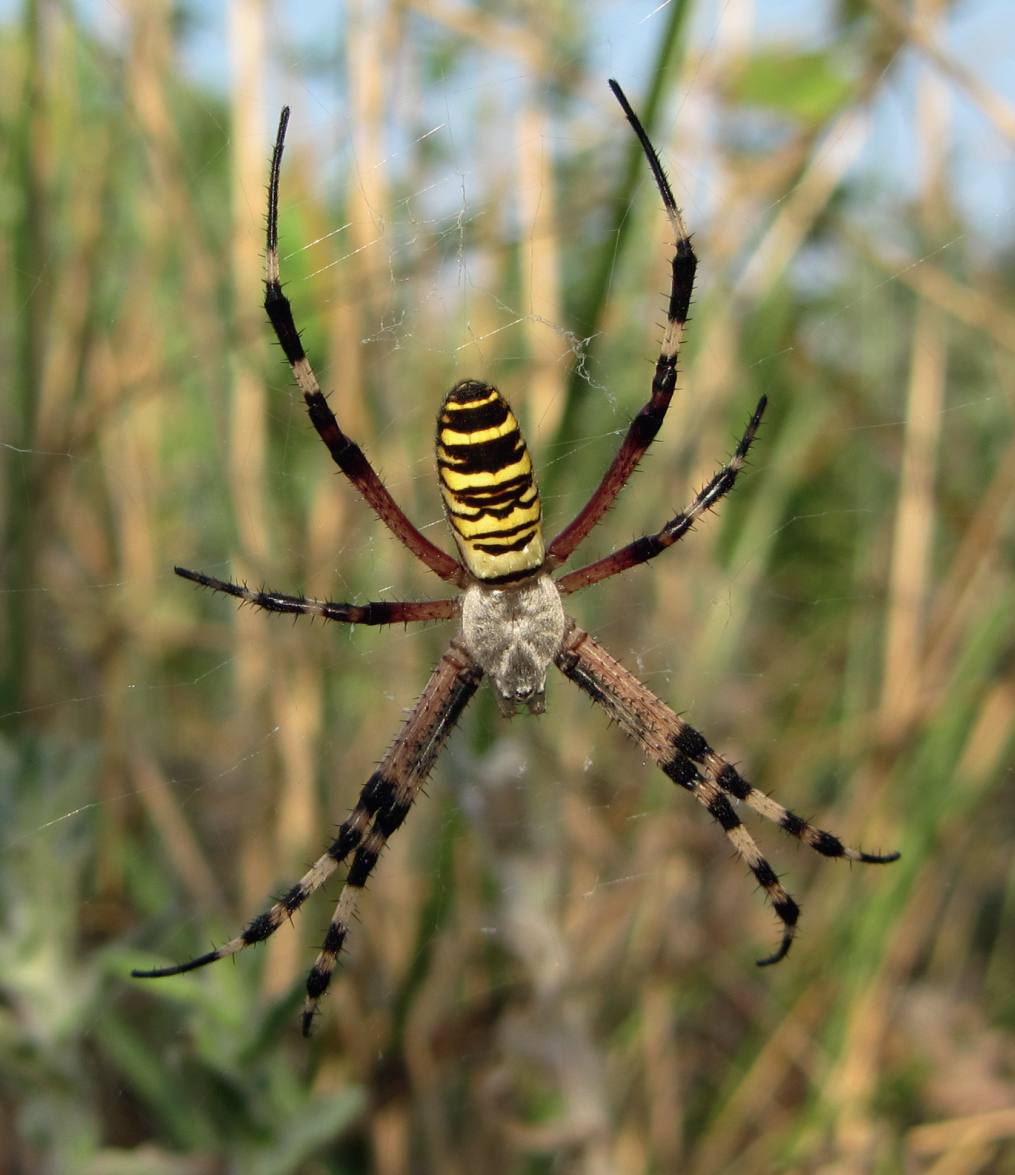
*Argiope bruennichi* female.

In contrast to the stereotype of male eagerness to mate with multiple females (Bateman 1948; Trivers 1972), monogynous males in various species actively limit themselves to mating with a single female in their lifetime (Hosken et al. 2009; Schneider and Fromhage 2010). As monogyny eliminates the usual trade-off between investment in the current mating versus investment in future matings, it allows for the evolution of strikingly counter-intuitive male traits. For example, in deep-sea ceratioid anglerfishes, the male grasps the much larger female with his teeth and then, by a gradual fusion of tissues and circulatory systems, transforms himself into a permanent attachment of her body (Pietsch 2005). In honey bees, stingless bees and certain ants, males bring about their own death by breaking off their abdomen during mating (Boomsma et al. 2005). Similarly, breakage of male copulatory organs during mating, followed by male death or functional sterility, is common in many spider species (Schneider and Fromhage 2010). Three types of adaptive benefits have been hypothesized to favor the evolution of monogyny. Monogynous males might be able to increase (1) their mate's reproductive output (the paternal investment hypothesis; Buskirk et al. 1984), or (2) their probability of mating (the mating hypothesis; Boomsma et al. 2005), or (3) they might be able to increase their paternity share at the expense of any other (potential) mates of the same female (Yamamura and Tsuji 1989; Fromhage et al. 2005). In spiders, which are the best studied group in this respect, substantial empirical support exists only for the paternity hypothesis (Schneider and Fromhage 2010). For example, *Latrodectus hasselti* males can increase their paternity by inducing the female to cannibalize them (Andrade 1996), *Argiope aurantia* males function as whole-body mating plugs after dying spontaneously during copulation (Foellmer and Fairbairn 2003), and in various other species, male genital fragments function as mating plugs (Fromhage and Schneider 2006; Nessler et al. 2007; Kuntner et al. 2009). Theoretical models suggest that monogyny should evolve as a consequence of a male-biased sex ratio (Fromhage et al. 2005, 2008). This prediction is consistent with data from *L. hasselti* (Andrade 1996), *A. aurantia* (Foellmer and Fairbairn 2005)*, Nephila clavata* (Miyashita 1993), *N. clavipes* (Christenson et al. 1985; Christenson 1989), and *Nephila fenestrata* (Fromhage et al. 2007), as well as being supported by a comparative study across aranoid spiders (Miller 2007). The models also predict that in certain areas of parameter space, neither monogyny nor its alternative (which might be either polygyny or bigyny) is evolutionarily stable, leading to the stable coexistence of strategies under negative frequency-dependent selection (i.e., a mixed Evolutionary Stable Strategy [ESS]). Biologically, such a mixed ESS could manifest itself either as a genetic polymorphism or as a genotype that produces probabilistic behavior (Maynard Smith 1982). If there is local variation in the success of alternative behaviors, however, and if individuals are capable to adjust their behavior flexibly, then we may instead expect the evolution of a conditional strategy. In the present context, this would mean that males should be monogynous in some situations, but not in others. An ideal species to test these ideas is the wasp spider *Argiope bruennichi* (Fig. 1), in which monogyny and bigyny coexist as alternative behaviors (Welke et al. 2012; Zimmer et al. 2012). *A. bruennichi* males can perform a maximum of two copulations in their life, one with each of their paired mating organs (pedipalps). Each copulation is associated with a risk of cannibalism. Males can increase their survival by attempting to escape earlier, leading to a negative relationship between copulation duration and cannibalism. For example, Nessler et al. (2009) showed that males raised in the absence of female pheromones copulated for shorter and were more likely to survive their first copulation. If a male dies during his first copulation, we refer to this outcome as monogyny type 1. If a male survives his first copulation, he may either use his remaining pedipalp to mate again with the same female (monogyny type 2), or he may leave to search for another female (bigyny; here defined as the attempt, not the act, of finding a second female). Males' second copulations always end in cannibalism, without any attempt of escape. By not attempting to escape, a male can maintain genital contact for longer, including for several seconds after the female has already grasped him, attempting to pull him away. In *A. bruennichi*, the females' paired genital openings are subject to the constraint of ipsilateral insemination, meaning that the left opening can only be inseminated by a left pedipalp and vice versa. A single short copulation suffices to ensure full female fertility (Schneider et al. 2005). If a female mates with more than one male, paternity depends on the males' relative copulation durations (Schneider et al. 2006). As used genital openings are often plugged by a fragment of the male's pedipalp, copulations into used openings have a low probability of sperm transfer (Uhl et al. 2007). Moreover, males can distinguish between virgin and non-virgin females, as well as between females of different sizes (Schulte et al. 2010). This situation presents a male with several options when encountering a female. First, should he copulate with her or should he search for a different female instead? Second, if he decides to copulate, how long should he aim to copulate in order to balance the benefit of sperm transfer against the risk of cannibalism? Finally, if he survives his first copulation, should he perform his second (and final) copulation with the same female or should he leave to search for a different female instead? The answers to these questions might depend on the state of the male and female involved, as well as on the progression of the mating season. Here, we use a stochastic dynamic game model to analyze this problem. We predict within-population patterns of time-and-context-dependent mating decisions in *A. bruennichi*. We also assess how these patterns depend on ecological variables that might vary across populations or across similar species.

## The Model

### Population composition

We assume an infinite population in which initially there are *R* males per female. All individuals are initially subadult. We express numbers in relation to the number of subadult females initially present, which is arbitrarily defined as 
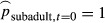
. For example, to state that there are *S* potential web-sites (henceforth, “sites”) per subadult female initially present, we say that there are *S* sites. At any given time, each site can be inhabited by no more than one female. The mating season is divided into *t*_max_ time steps. Each step corresponds to the time needed by a male to move from one site to another. For an overview of parameter values and notation, see Table 1. For mathematical details, see Appendix and supplementary tables.

**Table 1:** Parameters and variables

Parameter	Explanation; default value
*plug*	Probability that a copulation does not involve any sperm transfer, given the used genital opening has been used before; 0.8
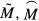	Maturation rate of males and females, respectively; 0.5, 1
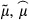	Mortality rate of males and females, respectively; 0.02, 0.02
*Δ*	Time for which a male can extend a copulation beyond the female's attack by not attempting to escape; 10
*R*	Sex ratio, males/females; 1
*S*	Web-sites available per female; 1
*α*_virgin_*, α*_mated_	Coefficients scaling the rates at which males are attracted by virgin or mated females, respectively; 5, 1
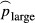	Proportion of all female that are large; 0.5
*fecundity*_small_	Factor by which a large female produces more eggs than a small female; 0.5
*t*_max_	Number of time steps of a mating season; 10
**Variable**	**Explanation**
*c*	Attempted copulation duration
*x*	Units of transferred sperm
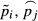	Frequencies of males in state *i* and females in state *j*, respectively, expressed in relation to the initial number of subadult females, 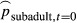
*t*	The current time step, where *t*_max_ marks the end of the breeding season
*used*	State of a particular genital opening (*used* = 1 and *use*d = 0 for used or unused openings, respectively)

### Life history

Subadult males and females moult to adulthood with sex-specific probabilities 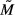 and 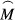, respectively, in each time step. We use default values 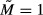 and 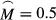 (but see Fig. 4) to generate protandry where all males mature at the beginning of the mating season, whereas females continue to mature as the season progresses. Males and females are subject to sex-specific mortality probabilities 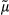 and 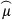 in each time step. Females vary in fecundity, which can be detected by males because it reflects female body size. For simplicity, we consider only two size classes, where large females make up a proportion 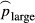 of all females, and small females lay a proportion *fecundity*_small_ of the eggs produced by large females. Oviposition occurs at the end of the mating season.

### Mate search

In each time step, all adult males simultaneously move to a new site, such that each male's probability of arriving at a given randomly chosen site is 1/*S*. However, because females emit male-attracting pheromones (Chinta et al. 2010), a site inhabited by an adult female is *a* times as likely to be visited as an empty site, where *a* takes values *a*_virgin_, and *a*_mated_ for virgin and mated females, respectively.

### Mating

Males perform a maximum of two copulations in their lifetime; one with each pedipalp. The mating pattern is ipsilateral, that is, a male's right pedipalp fits only into a female's right copulatory opening, and the left pedipalp fits only into the left opening. If a male encounters an adult female, he attempts to copulate with her for *c* ∈ {0, 1, 2…10} seconds, as prescribed by his strategy. Here, the value *c* = 0 is interpreted such that the male does not attempt to copulate and instead moves to another site in the next time step. If *c* > 0, he copulates. During copulation, the male is at risk of being attacked by the female. Based on the observation that *A. bruennichi* females generally attack within the first 10 sec of copulation (Schneider et al. 2006), we let a male's survival probability decrease linearly from 1 to 0 within 10 sec from the onset of copulation. If 0 < *c* < 10, the male may either get killed or he may escape after *c* seconds; whatever happens first. If a male attempts to escape, this terminates copulation immediately. By contrast, if he does not attempt to escape, he faces certain death, but can extend his copulation for *Δ* seconds after the female's attack. If a virgin male survives his first copulation, he may immediately mate again with the same female for *c*_2_ seconds, as prescribed by his strategy. If a male copulates into a previously used genital opening, sperm transfer fails with probability *plug*, because the opening may be obstructed by a mating plug placed by the predecessor. Otherwise, sperm transfer is a linear function of copulation duration, with one unit of sperm being transferred per second.

### Mating strategies

A male's strategy specifies how many seconds (integer values between 0 and 10) a male should attempt to copulate in any given situation. Situations are defined by combinations of the following factors: male mating status (virgin or not); female mating status (virgin or not) upon the male's arrival; female size (large or small); mating history between the focal pair (has the male already mated with this female in the present time step?); time of season (*t*). Considering only biologically meaningful combinations of these factors (e.g., a male cannot be virgin if the pair have just mated), this defines 12*t*_max_-8 different situations for which a male's strategy must specify an action.

### Male reproductive success

Paternity is determined by relative quantities of transferred sperm according to a ‘fair raffle’. A male's reproductive success is given by the summed fecundity of his mate(s) that survive to the end of the mating season, weighted by his paternity.

### State variables

Adult males can be in either of two states: virgin or mated. The mated category includes only males that have previously used exactly one of their two pedipalps, and that are hence capable to perform one more copulation in the future. Males that have already used both pedipalps can no longer participate in reproduction and are hence disregarded. Adult females are described by three variables: (1) status of the paired genital openings (“virgin” when both openings are unused; “half-plugged” when one opening has been used; “fully plugged” when both have been used), (2) amount of stored sperm, and (3) body size (large or small; assumed to be fixed over a female's life). For simplicity, to limit the classes of females that need to be tracked separately, we do not explicitly model the possibility that mating plug placement fails completely, in addition to the functionally very similar possibility that a mating plug fails to work. Males have incomplete knowledge of a female's state. They can detect a female's size and whether or not she is a virgin, but no further details about her mating history. As our formulation cannot accommodate a realistic female size distribution, we assume by default (but see Fig. 6) that half of the females are “large” 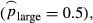 each producing twice as many eggs as “small” females (*fecundity*_small_ = 0.5). This fecundity difference is within the natural range of *A. bruennichi*.

### Forward computation

All males in the population use the same strategy, called the resident strategy. For any resident strategy, we can follow the course of a mating season by writing down recursions describing how numbers of individuals in different states change from one time-step to the next. For example, if there are *p* subadult individuals of a given sex at time *t*, then at time *t* + 1, there are


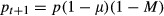


subadult individuals of that sex.

### Dynamic programming

We use dynamic programming (Houston and McNamara 1999) to calculate the reproductive value *V* (defined as the expected number of offspring produced from time *t* onwards) of males at any time *t*. For example, the reproductive value of a virgin male upon entering time step *t* is given by


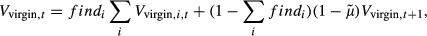


where *find*_i_ is the probability of finding a type *i* female, *V*_virgin*,i,t*_ is the reproductive value upon finding such a female, and the term on the right hand side is the fitness gain from finding no female in the present time step. The details of calculating *V*_virgin*,i,t*_ (and its equivalent for mated males) are given in Tables S6–14. To find an optimal mutant strategy, we first consider the final time step, and then work backwards from there until the first time step. For any situation a male may face in the final time step, we calculate the expected reproductive success to be gained by any action. Actions maximizing reproductive success are assigned as part of the mutant strategy. We then repeat this procedure for the penultimate time step and so on, always assuming that the mutant will behave optimally in future time steps. Once the optimal mutant strategy is defined for all time steps, we assign it as the new resident to be used in forward computations as described above. Through alternating cycles of forward and backward computations, this procedure arrives at a strategy that cannot be invaded by any mutant. To ensure that convergence always occurs, we use Houston and McNamara's (1999, p. 191) method to include errors in decision-making, using the function 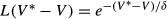 to describe how costlier errors occur less frequently than near-optimal actions. Here, V* is the reproductive value associated with the optimal action, *V* is the reproductive value associated with any action of interest, and the coefficient *δ* = 0.1, chosen by trial and error, sets error frequency to the minimal level required to ensure convergence for all cases.

## Results

**Figure 2:**
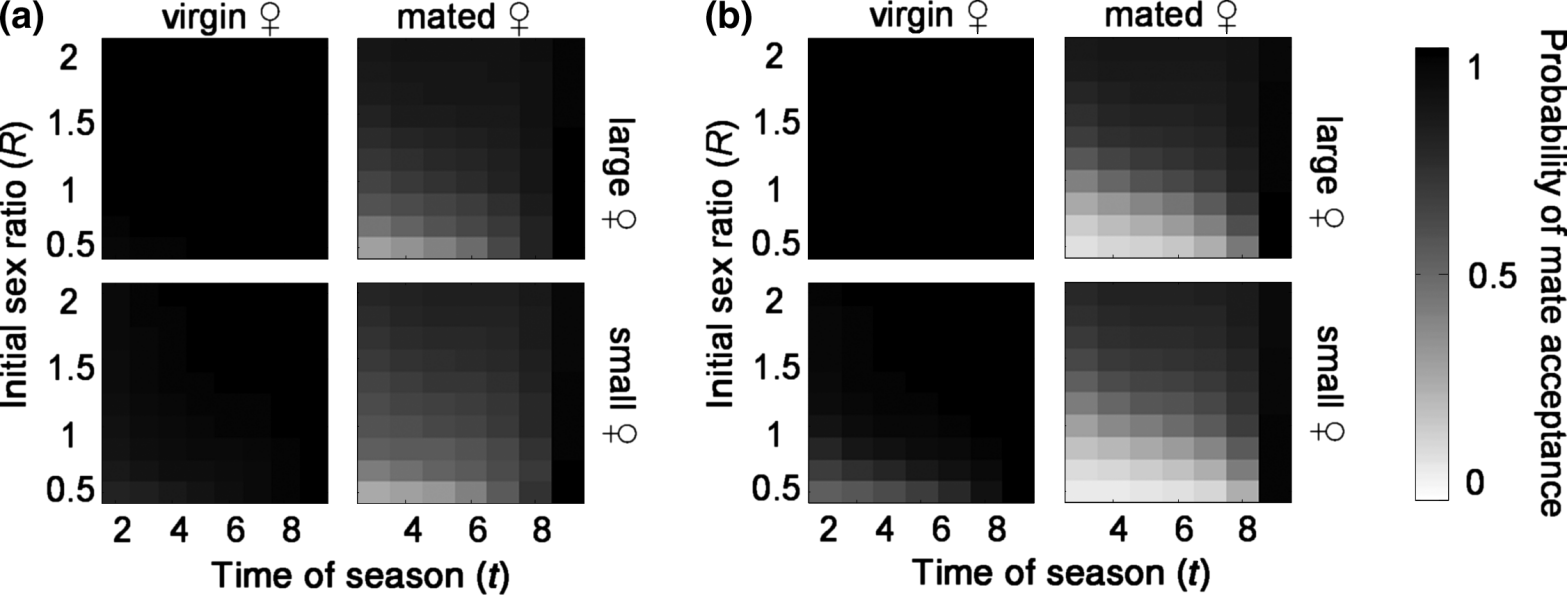
Effect of the initial sex ratio on mate acceptance by virgin (a) and mated (b) males at different times of season. Shading represents the probability that a male decides to mate with (i.e., does not reject) females of different types (size, mating status).

Males in our model almost never reject virgin females, but they sometimes reject mated females (Fig. 2), especially if the sex ratio is female-biased. Mated males (Fig. 2a) are choosier (i.e., more likely to reject a given female) than virgin males (Fig. 2b). Male choosiness decreases toward the end of the mating season, until no females are rejected in the final time step.

**Figure 3:**
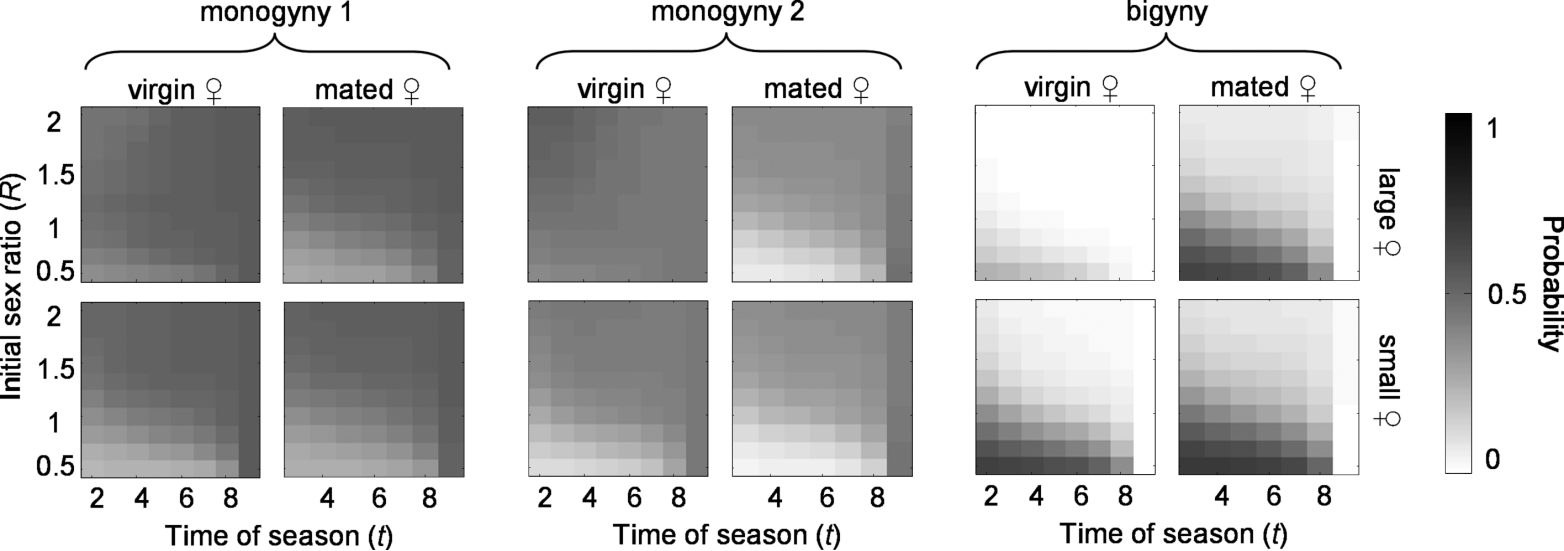
Effect of the initial sex ratio on virgin males' mating outcomes at different times of season. Once a virgin male decides to mate, the outcome may be cannibalism during his first mating (monogyny 1), or a double mating (monogyny 2), or the male may leave the female after mating once (bigyny). The probabilities of these outcomes add up to one.

Monogynous behavior occurs most commonly with large virgin females (Fig. 3), whereas bigyny occurs mostly in matings with small and/or already mated females (Fig. 3). Bigyny becomes rarer toward the end of the mating season, until no bigyny occurs in the final time step. Monogyny of types 1 and 2 generally occurs at similar frequencies, although a slight bias toward type 1 is apparent for matings with mated females (Fig. 3). Because of this similarity, and because the total probability of monogyny is implicit in the probability of bigyny (since Prob (monogyny type 1) + Prob (monogyny type 2) + Prob (bigyny) = 1), we present further results only in terms of bigyny.

**Figure 4:**
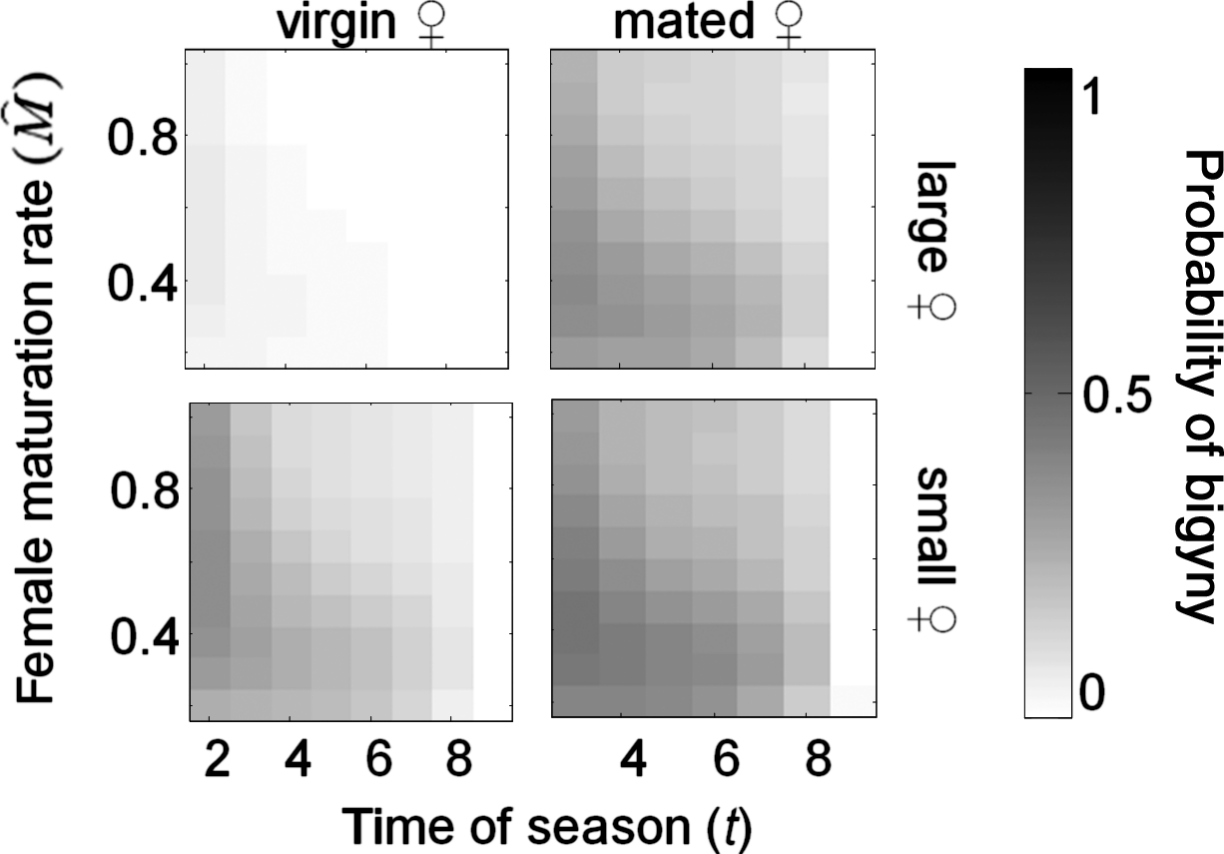
Effect of female maturation rate on the probability that a male leaves a female after his first copulation (bigyny), for different types of females and times of season. As all males mature in the first time step 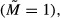 lower probabilities 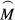 of female maturation per time step correspond to greater degrees of protandry.

**Figure 5:**
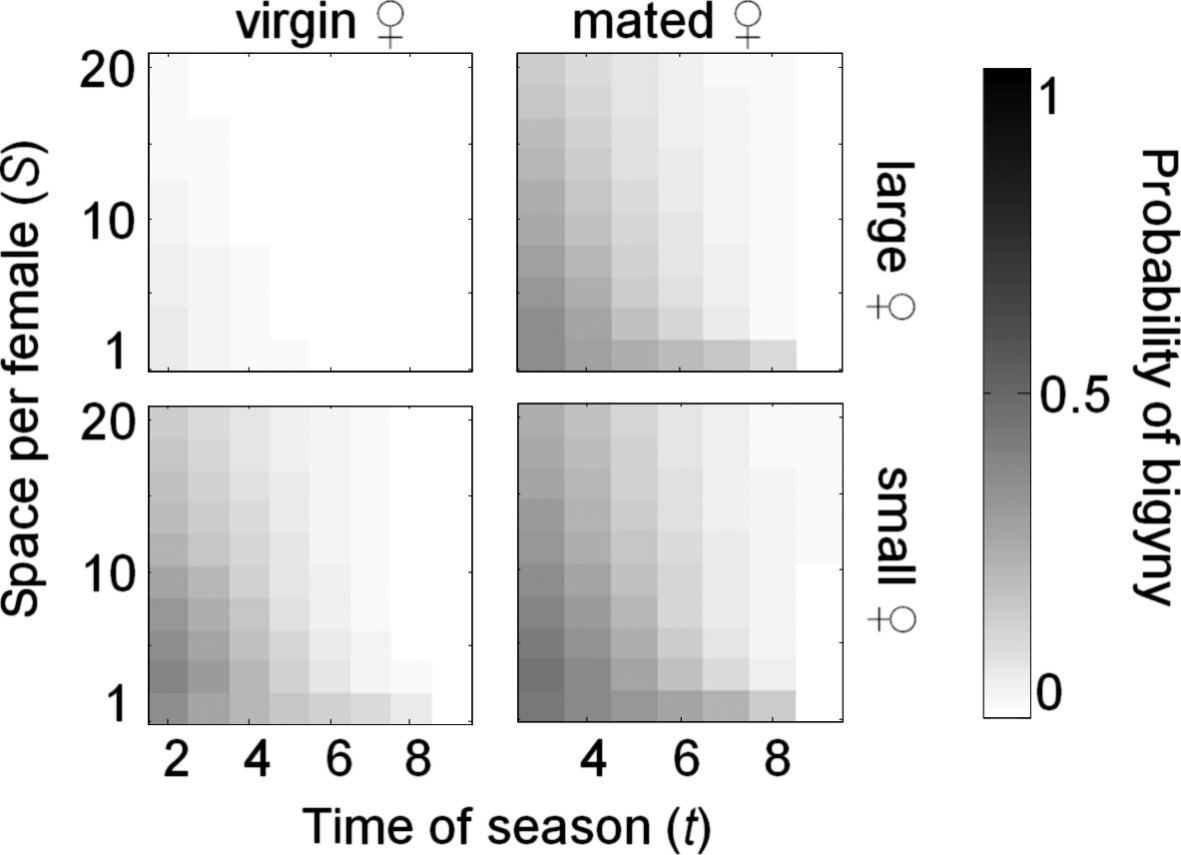
Effect of population density on the probability that a male leaves a female after his first mating (bigyny), for different types of females and times of season.

**Figure 6:**
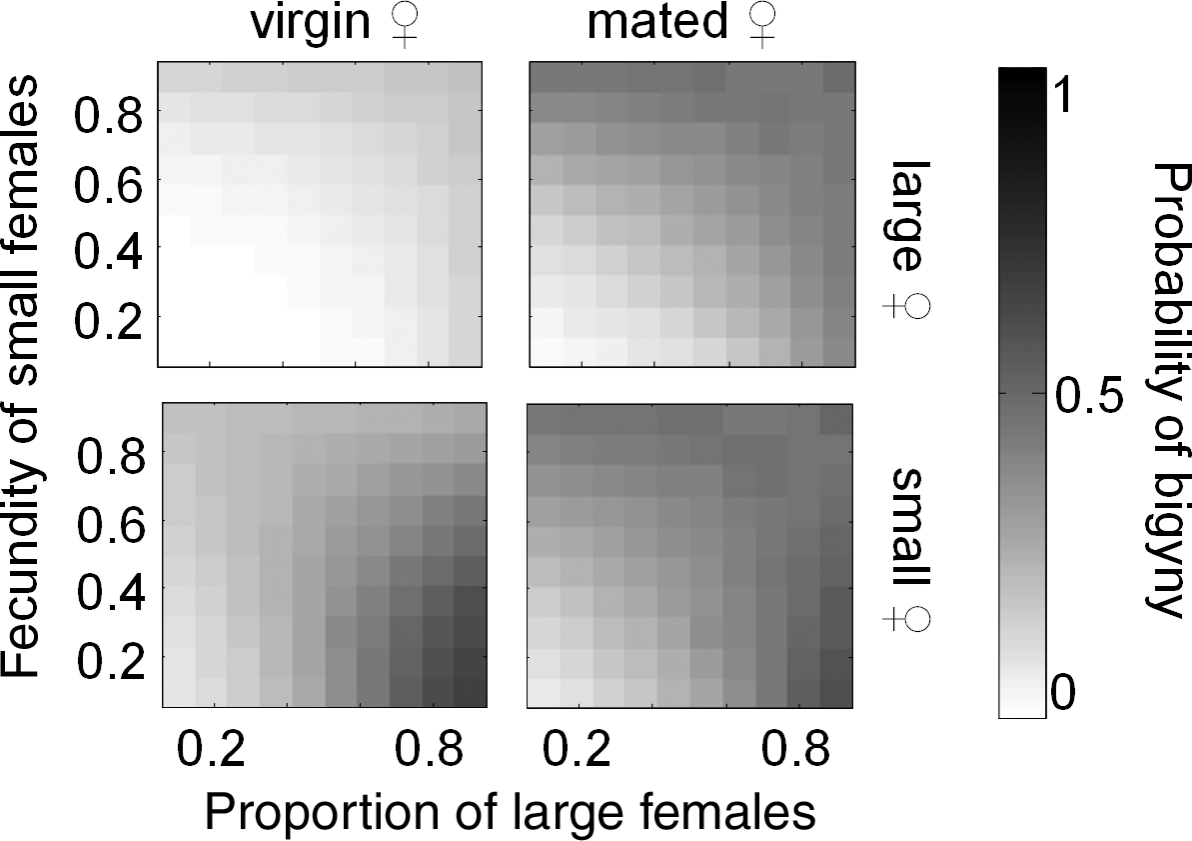
Proportion of males leaving different types of females after the first mating (bigyny), during the whole season, in parameter space of the proportion of large females, and the fecundity of small females.

A high rate of female maturation (which is equivalent to a low degree of protandry; males are always assumed to mature at a high rate) tends to decrease the probability of bigyny (Fig. 4). High population density (i.e., a low number *S* of potential web-sites per female) has a weak positive effect on the probability of bigyny (Fig. 5). The occurrence of bigyny in matings with large virgin females is generally low, and especially so if large females are rare and the fecundity disadvantage of small females is severe (Fig. 6).

## Discussion

Here, we show that males can benefit from choosing strategically between mating with either one or two females in their lifetime. The optimal choice depends largely on female quality, as well as on seasonality.

Previous theory indicated that obligate monogyny (an inflexible mating strategy not taking into account local conditions) generally requires a male-biased sex ratio to evolve (Fromhage et al. 2005, 2008). Although our present model confirms a positive effect of the sex ratio on the frequency of monogynous matings, it also shows that a male-bias is not necessary for the evolution of conditional monogyny (Fig. 3). The intuitive reason for this is that a male can more easily benefit from sacrificing his life for a particularly valuable female, rather than for any random female he meets. In other words, if some females offer above-average reproductive returns (as is true for large females in our model, especially if they are rare and their fecundity advantage is strong; Fig. 6), it can pay for males to make a terminal investment when mating with such females.

The evolution of male mate choice in sequential encounter scenarios is generally considered difficult because it involves rejecting some reproductive opportunities before it is known whether a better opportunity will ever arise (Barry and Kokko 2010). Nevertheless, consistent with empirical findings in *A. bruennichi* (Schulte et al. 2010), our model predicts that males should be choosy, often rejecting to mate with already mated females (Fig. 2). Choosiness is predicted to be stronger in mated males (Fig. 2), who face higher opportunity costs of mate acceptance (i.e., certain death) than do virgin males. This prediction, however, conflicts with experimental findings in *A. keyserlingi* (Gaskett et al. 2004), where virgin but not mated males preferred virgin over mated females in binary choice situations. This discrepancy may be explained by confounding factors that are empirically associated with male mating status: whereas we have assumed here that virgin and mated males have the same mate search ability, mated males in nature have often lost some of their legs during copulation (Fromhage et al. 2003; Gaskett et al. 2004). This may impair their mobility and sensory acuity (spider legs are covered with sensory hairs; Foelix 1996), potentially explaining their reduced choosiness. Another potential confounding factor is that mated males may have been older than virgin males. In recent binary choice experiments using *A. bruennichi*, both confounding factors were controlled: only mated males with eight legs were used and age was varied in virgin and mated males. No differences in choosiness were found between virgin and mated males, although male age (independent of mating status) had an influence on their choosiness in that older males showed a stronger preference for heavy females than did younger males (J. M. Schneider, A. Gatz, K. Sauerland, S. M. Zimmer, unpubl.). These issues deserve further empirical investigation.

Our model predicts that both types of monogyny should occur mostly with large, virgin females, whereas bigyny should be most common with small, mated females (Fig. 3). Consistent with this prediction, a recent field study in *A. bruennichi* found that bigyny was more common in matings with small females, and that monogyny type 2 occurred almost exclusively in matings with virgin females (in 16 of 17 cases, although the overall effect of female mating status on male mating tactics was non-significant) (Welke et al. 2012). However, the same study found no support for our predicted bias toward bigyny in matings with mated females; instead, bigyny and monogyny type 1 were similarly common among matings with mated females. This discrepancy between theory and data may be due to mated females being particularly aggressive, forcing more males into the fate of monogyny type 1 than would be the case under our assumption of uniformous female behavior.

Monogyny type 1 occurs when a male is killed by the female during his first mating. Whether or not this happens depends on his attempted copulation duration: with every additional second he can transfer more sperm, but at a cost of reducing his survival chances. As the conditions favoring a high sperm investment in the first copulation are similar to the conditions favoring repeated mating with the same female (namely, a high value of the focal mating opportunity in terms of female size and virginity), both types of monogyny are predicted to occur at similar frequencies (Fig. 3). In contrast, Welke et al. (2012) found monogyny type 1 to be twice as common as type 2, suggesting that males in nature may be less able to avoid cannibalism than we have assumed in our model.

*Argiope bruennichi* males can also vary their mating tactics in response to environmental cues: Nessler et al. (2009) found that males raised in the presence of virgin females' pheromones were more likely to get cannibalized during their first copulation (=monogyny type 1) than control males. It is not entirely clear, however, to what natural context this result relates: for example, males might use female pheromones to assess seasonal timing or population density (see Zimmer et al. 2012 for a field study showing a correlation between male activity and virgin females' density). If control males in Nessler et al.'s study perceived themselves to be at the beginning of the season (before the peak of pheromone production), their increased survival fits our prediction that bigyny should be common early in the season (Figs. 2–4). However, this prediction was not supported by field data (Welke et al. 2012). On the other hand, if control males in Nessler et al.'s study perceived themselves to live at a low population density, their increased survival runs contrary to our prediction of low population density favoring monogyny (Fig. 5). In view of these inconclusive interpretations, further work appears necessary to clarify whether the patterns found by Nessler et al. (2009) are in fact adaptive and relevant in the wild.

A largely unexplored line of inquiry is the study of demography in relation to mating tactics at a geographic scale, which would be interesting in species with wide distribution ranges such as *A. bruennichi* or *A. keyserlingi*. This approach would allow testing our predictions that bigyny should be more common in dense populations (Fig. 5) with a high degree of protandry (Fig. 4).

Our model suggests that conditional monogyny can evolve under broad conditions, justifying a careful search for its existence beyond the genus *Argiope*. Promising candidate species are those where monogyny has been observed in the absence of strict morphological constraints that would make it obligatory; this includes the spider genus *Latrodectus* and the family Nephilidae (Schneider and Fromhage 2010). We conclude by noting that conditional monogyny may also be a plausible intermediate stage in lineages that eventually evolve obligate monogyny.

## Appendix

The described mate search process implies that in any given time step, a proportion

of adult males move to a site inhabited by a type *i* female, where  is the number of type *i* females at time *t*. Multiplying by the number  of searching males and dividing by the number  of type *i* females, we obtain the mean number

of encountered males per type *i* female. Thus, we can infer from a Poisson distribution that a proportion

of type *i* females encounters at least one male. With the additional assumption that only the first male to reach a given female in a given time step gets an opportunity to mate, it follows that males obtain mating opportunities with type *i* females with probability

We define

as the probability that a male decides to mate in a given mating opportunity. The subscript *k* distinguishes between the possibility that this is the first (*k* = 1) or second (*k* = 2) mating between the focal pair. Assigning the value *used* = 1 to previously used genital openings and *used* = 0 to unused genital openings, a female's probability of receiving *x* units of sperm in a mating with attempted duration *c* is given by

Here, the first line refers to males not attempting or not succeeding to mate (because of a mating plug); the second line refers to males attempting to escape after mating for less than *x* seconds; the third line refers to copulation durations too long to be reached even by a male who does not attempt to escape; the fourth line refers to copulation durations shorter than the minimum achieved by a male not attempting to escape; the fifth line refers to copulation durations achieved by a male not attempting to escape, given that he is attacked at some particular time; the sixth line refers to copulation durations resulting from a male's unsuccessful attempt to escape; and the seventh line refers to matings in which the male is attacked at the very second he attempts to escape.

If a male attempts to copulate for *c* seconds, his probability of getting killed is given by

and his conditional probability of getting killed, given that the copulation lasts for *x* seconds, is given by

Recursions for the numbers of virgin and mated males and females can be written as

(X)

where the elements *v*_*h*,*t*_ are as specified in Tables S1–5.

Consider a female of size *i* that has a certain plugging status (*v* for virgin; *h* for half-plugged; *f* for fully plugged) when entering time step *t*, and that may have a different plugging status when entering time step *t* + 1. By following female mating histories through time using forward computations, we can calculate the probability θ(*X*) that the female receives a total of *X* sperm units over the entire season (excluding the focal time step), depending on her plugging status when entering and leaving the focal time step. We write this probability as θ_vh,i_(*X*) for size *i* females whose mating status changes from *v* to *h* in the focal time step, as θ_vf,i_(*X*) for females whose mating status changes from *v* to *f* in the focal time step, and so on. These probabilities are needed to calculate the expected reproductive success from a given copulation, which depends on the total amount of sperm that the focal male's sperm face in sperm competition (Tables S6–14).

## Supporting Information

**Table S1.** Recursion for virgin males.

**Table S2.** Recursion for mated males.

**Table S3.** Recursion for virgin females of size *i*.

**Table S4.** Recursion for half-plugged females of size *i* with *X* units of stored sperm.

**Table S5.** Recursion for fully plugged females of size *i* with *X* units of stored sperm.

**Table S6.** Reproductive value  of a male who (1) has entered the present time step as a virgin male, (2) has encountered a virgin female of size i, (3) has already copulated with this female once, transferring *x*_1_ units of sperm, and (4) is about to use his second pedipalp to copulate into the female's second opening for *c*_2_ seconds.

**Table S7.** Reproductive value  of a virgin male who has encountered a virgin female of type i and is about to copulate with her for the first time, for *c*_1_ seconds.

**Table S8.** Reproductive value  of a male who (1) has entered the present time step as a virgin male, (2) has encountered a half-plugged female of type *i* (3) has already copulated with this female once, for *c*_1_ seconds, and (4) is about to use his second pedipalp to copulate into the female's second opening, for *c*_2_ seconds.

**Table S9.** Reproductive value  of a virgin male who has encountered a half-plugged female of type *i* and is about to copulate with her for the first time, for *c*_1_ seconds.

**Table S10.** Reproductive value  of a male who (1) has entered the present time step as a virgin male, (2) has encountered a fully plugged female of type i and (3) has already copulated with this female once, for *c*_1_ seconds, and (4) is about to use his second pedipalp to copulate into the female's second opening for *c*_2_ seconds.

**Table S11.** Reproductive value  of a virgin male who has encountered a fully plugged female of type i and is about to copulate with her for the first time, for *c*_1_ seconds.

**Table S12.** Reproductive value  of a mated male who has encountered a virgin female of type i and is about to use his remaining pedipalp to copulate with her for *c*_1_ seconds.

**Table S13.** Reproductive value  of a mated male who has encountered a half-plugged female of type i and is about to use his remaining pedipalp to copulate with her for *c*_1_ seconds.

**Table S14.** Reproductive value of a male who has encountered a fully plugged female of type i and is about to use his remaining pedipalp to copulate with her for *c*_1_ seconds.
