# Supplementary material for: A mate to die for? A model of conditional monogyny in cannibalistic spiders
Source: Ecol Evol. 2012 Sep 13;2(10):2577–87. doi: 10.1002/ece3.372 (PMC3492783; doi:10.1002/ece3.372)
Supplement: Supplementary file 1 [file ece30002-2577-SD1.doc]

Supplementary table 1. Recursion for virgin males.

|  | Virgin males survive to the next time step. |
| --- | --- |
|  | Virgin males become non-virgin because they mate with females of type i. |
|  | Subadult males mature at rate M and join the pool of virgin males (given they survive, with probability $1-\tilde{\mu}$). |

Supplementary table 2. Recursion for mated males.

|  | Mated males survive to the next time step. |
| --- | --- |
|  | Mated males make their second copulation with a type i female. |
|  | Virgin males mate once with a type i female and then leave, thus becoming mated males. |

Supplementary table 3. Recursion for virgin females of size i.

|  | Virgin females survive to the next time step. |
| --- | --- |
|  | Virgin females become non-virgin through mating with a virgin male. |
|  | Virgin females become non-virgin through mating with a mated male. |
|  | Subadult females mature at rate and join the pool of virgin females (given they survive, with probability . |

Supplementary table 4. Recursion for half-plugged females of size i with *X* units of stored sperm.

|  | Females in the focal category survive to the next time step. |
| --- | --- |
|  | Females leave the focal category by mating with a virgin male (in his first attempt). Because the male has probability ½ of selecting the plugged opening, his attempt fails with probability *plug*/2. |
|  | Females leave the focal category by mating with a virgin male (only in his second attempt, after his 1^st^ attempt failed). |
|  | Females leave the focal category by mating with a mated male. |
|  | Virgin females, if they survive (with probability) enter the focal category by mating with a virgin male who performs a single copulation of *X* seconds, but who does not perform a second copulation. |
|  | Virgin females enter the focal category by mating with a mated male, who performs a single copulation of *X* seconds, but does not perform a second copulation. |
|  | Half-plugged females that have previously copulated for a total duration of *y* (<*X*) enter the focal category by receiving a copulation of *X-y* seconds into the already used opening, performed by a virgin male in his first attempt. The male performs no second attempt with the same female. |
|  | Half-plugged females that have previously copulated for a total duration of *y* (<*X*) enter the focal category by receiving an additional copulation of *X-y* seconds into the already used opening, performed by a mated male. |

Supplementary table 5. Recursion for fully plugged females of size i with X units of stored sperm.

|  | Females in the focal category survive to the next time step. |
| --- | --- |
|  | Females leave the focal category by mating with a virgin male (in his first attempt). |
|  | Females leave the focal category by mating with a virgin male (only in his second attempt). |
|  | Females leave the focal category by mating with a mated male. |
|  | Virgin females enter the focal category by mating with a virgin male, who survives a first copulation of c seconds, followed by a second copulation of *X-c* seconds. |
|  | Half-plugged females that have previously copulated for a total duration of y (<*X*) enter the focal category by receiving a complementary mating (i.e., a mating in the as yet unused genital opening of duration *X-y*) in a virgin male’s first attempt. This attempt is not immediately followed by another successful attempt. This formulation accounts for the probability 1/2 that the correct (unused) opening is targeted, as well as for the probability (1-*cann*)*mate_2_*(1-*plug*)) that the male survives and immediately mates again. |
|  | Half-plugged females that have previously copulated for a total duration of *y* (<*X*) enter the focal category by receiving a complementary mating of duration *X-y* during a virgin male’s second attempt (after his first attempt failed because of the plug in the other opening). |
|  | Half-plugged females that have previously copulated for a total duration of y (<*X*) enter the focal category by receiving a complementary mating of duration *X-y*, performed by a mated male. |
|  | Half-plugged females that have previously copulated for a total duration of *y* (<*X*) enter the focal category by receiving two additional copulations of durations *z*=1..*X*-1 and *X-y-z*, performed by a virgin male. The male uses the unused opening first. |
|  | As above, except that the male uses the used opening first. |
|  | Fully plugged females that have previously copulated for a total duration of *y* (<*X*) enter the focal category by receiving an additional copulation of duration *X-y*, performed by a virgin male in his first attempt (which is his only successful attempt with this female). |
|  | Fully plugged females that have previously copulated for a total duration of y (<*X*) enter the focal category by receiving an additional copulation of duration *X-y*, performed by a virgin male in his second attempt (which is his only successful attempt with this female). |
|  | Fully plugged females that have previously copulated for a total duration of *y* (<*X*) enter the focal category by receiving two additional copulations of durations *c*_1_ and *X-y-c*_1_, performed by a virgin male. |
|  | Fully plugged females that have previously copulated for a total duration of *y* (<*X*) enter the focal category by receiving an additional copulation of duration *X-y*, performed by a mated male. |

Supplementary table 6. Reproductive value $V_{virgin,virgin,2,t}\mathop{=\max}\limits_{c_{2}} \left(\sum_{j} v_{j}\right)$ of a male who (1) has entered the present time step as a virgin male, (2) has encountered a virgin female of size i, (3) has already copulated with this female once, transferring *x*_1_ units of sperm, and (4) is about to use his second pedipalp to copulate into the female’s second opening for *c*_2_ seconds.

|  | The male remates with the focal female. |
| --- | --- |
| $v_{2}=(1-mate_{2})(1-\tilde{\mu})V_{mated,t+1}$ | The male does not remate with the focal female. Instead, he enters the following time step as a mated male (given he survives, with probability $1-\tilde{\mu}$). |

Supplementary table 7. Reproductive value $V_{virgin,virgin,1,t}\mathop{=\max}\limits_{c_{1}} \left(\sum_{j} v_{j}\right)$ of a virgin male who has encountered a virgin female of type i and is about to copulate with her for the first time, for *c*_1_ seconds.

|  | Fitness gain from a sequence of events where the male mates only once with a virgin female, transferring *x*_1_ units of sperm. |
| --- | --- |
|  | Fitness gain from a male’s first mating, given that he survives after copulating for *c*_1_ seconds, and then goes on to remate with the female. |
| $v_{3}=mate_{1}(1-cann\left\langle c_{1}\right\rangle)V_{virgin,virgin,2,t}$ | Fitness gain from surviving the 1^st^ mating with the focal female. |
| $v_{4}=(1-mate_{1})(1-\tilde{\mu})V_{virgin,t+1}$ | Fitness gain from deciding not to mate. |

Supplementary table 8. Reproductive value $V_{virgin,halfplugged,2,t}\mathop{=\max}\limits_{c_{2}} \left(\sum_{j} v_{j}\right)$ of a male who (1) has entered the present time step as a virgin male, (2) has encountered a half-plugged female of type *i* (3) has already copulated with this female once, for *c*_1_ seconds, and (4) is about to use his second pedipalp to copulate into the female’s second opening, for *c*_2_ seconds.

|  | Fitness gain from remating with the focal female, after his first copulation was into the female’s plugged opening (with probability 1/2), entailing no sperm transfer (with probability *plug*). |
| --- | --- |
|  | Fitness gain from remating with the focal female, given that the male’s first copulation was into the female’s plugged opening (with probability 1/2), but nevertheless involved sperm transfer (with probability 1-*plug*). |
|  | Fitness gain from remating with the focal female, given that the male’s first copulation was into the female’s unused opening (with probability 1/2), so that the second copulation is into a plugged opening. |
| $v_{4}=(1-mate_{2})(1-\tilde{\mu})V_{mated,t+1}$ | Fitness gain from deciding not to remate with the focal female. The mated male survives to the next time step (with probability $1-\tilde{\mu}$). |

Supplementary table 9. Reproductive value $V_{virgin,halfplugged,1,t}\mathop{=\max}\limits_{c_{1}} \left(\sum_{j} v_{j}\right)$ of a virgin male who has encountered a half-plugged female of type *i* and is about to copulate with her for the first time, for *c*_1_ seconds.

|  | Fitness gain from mating into the plugged opening, nevertheless transferring *x*_1_ units of sperm, and then being killed by the female. |
| --- | --- |
|  | Fitness gain from mating into the unplugged opening, transferring *x*_1_ units of sperm, and then being killed by the female. |
|  | Fitness gain from a male’s first mating, given that he survives after copulating for *c*_1_ seconds into the plugged opening (which involves sperm transfer with probability 1-*plug*), and given the male decides to remate with that female. |
|  | Fitness gain from a male’s first mating, given that he survives after copulating for *c*_1_ seconds into the plugged opening (which involves sperm transfer with probability 1-*plug*), and given the male decides not to remate with the female. |
|  | Fitness gain from a male’s first mating, given that he survives after copulating for *c*_1_ seconds into the unplugged opening, and given a second copulation (into the plugged opening, with sperm transfer *x*_2_) follows. |
| $v_{6}=mate_{1}(1-cann\left\langle c_{1}\right\rangle)V_{virgin,halfplugged,2,t}$ | Fitness gain from a male’s second mating with the same female. |
| $v_{7}=(1-mate_{1})(1-\tilde{\mu})V_{mated,t+1}$ | Fitness gain from deciding not to mate. |

Supplementary table 10. Reproductive value $V_{virgin,fullyplugged,2,t}\mathop{=\max}\limits_{c_{2}} \left(\sum_{j} v_{j}\right)$ of a male who (1) has entered the present time step as a virgin male, (2) has encountered a fully plugged female of type i and (3) has already copulated with this female once, for *c*_1_ seconds, and (4) is about to use his second pedipalp to copulate into the female’s second opening for *c*_2_ seconds.

|  | Fitness gain from remating with the focal female, given that the male’s first copulation involved no sperm transfer (with probability *plug*). |
| --- | --- |
|  | Fitness gain from remating with the focal female, given that the male’s first copulation involved sperm transfer (with probability 1-*plug*). |
| $v_{4}=(1-mate_{2})(1-\tilde{\mu})V_{mated,t+1}$ | Fitness gain from deciding not to remate with the focal female. The mated male survives to the next time step (with probability $1-\tilde{\mu}$). |

Supplementary table 11. Reproductive value $V_{virgin,fullyplugged,1,t}\mathop{=\max}\limits_{c_{1}} \left(\sum_{j} v_{j}\right)$ of a virgin male who has encountered a fully plugged female of type i and is about to copulate with her for the first time, for *c*_1_ seconds.

|  | Fitness gain from a sequence of events where the male transfers *x*_1_ units of sperm and is killed by the female. |
| --- | --- |
|  | Fitness gain from a male’s first mating, given that he survives a copulation of *c*_1_ seconds involving sperm transfer (with probability 1-*plug*), and a second copulation (with sperm transfer *x*_2_) follows. |
| $v_{4}=mate_{1}(1-cann\left\langle c_{1}\right\rangle)V_{virgin,fullyplugged,2,t}$ | Fitness gain from a male’s second mating with the same female. |
| $v_{5}=(1-mate_{1})(1-\tilde{\mu})V_{mated,t+1}$ | Fitness gain from deciding not to mate. |

Supplementary table 12. Reproductive value $V_{mated,virgin,t}\mathop{=\max}\limits_{c_{1}} \left(\sum_{j} v_{j}\right)$ of a mated male who has encountered a virgin female of type i and is about to use his remaining pedipalp to copulate with her for *c*_1_ seconds.

|  | Fitness gain from mating with the focal female. |
| --- | --- |
| $v_{2}=(1-mate_{1})(1-\tilde{\mu})V_{mated,t+1}$ | Fitness gain from deciding not to mate with the focal female. The male survives to the next time step with probability $1-\tilde{\mu}$. |

Supplementary table 13. Reproductive value $V_{mated,halfplugged,t}\mathop{=\max}\limits_{c_{1}} \left(\sum_{j} v_{j}\right)$ of a mated male who has encountered a half-plugged female of type i and is about to use his remaining pedipalp to copulate with her for *c*_1_ seconds.

|  | Fitness gain from mating with the focal female, given the copulation is into the plugged opening (with probability 1/2). |
| --- | --- |
|  | Fitness gain from mating with the focal female, given the copulation is into the unplugged opening (with probability 1/2). |
| $v_{4}=(1-mate_{2})(1-\tilde{\mu})V_{mated,t+1}$ | Fitness gain from deciding not to remate with the focal female. The male survives to the next time step with probability $1-\tilde{\mu}$. |

Supplementary table 14. Reproductive value $V_{mated,fullyplugged,t}\mathop{=\max}\limits_{c_{1}} \left(\sum_{j} v_{j}\right)$ of a male who has encountered a fully plugged female of type i and is about to use his remaining pedipalp to copulate with her for *c*_1_ seconds.

|  | Fitness gain from mating with the focal female. |
| --- | --- |
| $v_{4}=(1-mate_{2})(1-\tilde{\mu})V_{mated,t+1}$ | Fitness gain from deciding not to remate with the focal female. The male survives to the next time step with probability $1-\tilde{\mu}$. |
